# Metabolic Consequences of Gestational Cannabinoid Exposure

**DOI:** 10.3390/ijms22179528

**Published:** 2021-09-02

**Authors:** Kendrick Lee, Daniel B. Hardy

**Affiliations:** 1 Department of Physiology and Pharmacology, Schulich School of Medicine and Dentistry, Western University, 1151 Richmond Street, London, ON N6A 5C1, Canada; klee843@uwo.ca; 2 The Children’s Health Research Institute, The Lawson Health Research Institute, London, ON N6A 5C1, Canada; 3 Department of Obstetrics and Gynaecology, Schulich School of Medicine and Dentistry, The University of Western Ontario, 1151 Richmond Street, London, ON N6A 5C1, Canada

**Keywords:** Δ9-tetrahydrocannabinol, cannabidiol, intrauterine growth restriction, placental insufficiency, liver, heart, pancreas, glucose intolerance, dyslipidemia

## Abstract

Up to 20% of pregnant women ages 18–24 consume cannabis during pregnancy. Moreover, clinical studies indicate that cannabis consumption during pregnancy leads to fetal growth restriction (FGR), which is associated with an increased risk of obesity, type II diabetes (T2D), and cardiovascular disease in the offspring. This is of great concern considering that the concentration of Δ^9^- tetrahydrocannabinol (Δ9-THC), a major psychoactive component of cannabis, has doubled over the last decade and can readily cross the placenta and enter fetal circulation, with the potential to negatively impact fetal development via the endocannabinoid (eCB) system. Cannabis exposure in utero could also lead to FGR via placental insufficiency. In this review, we aim to examine current pre-clinical and clinical findings on the direct effects of exposure to cannabis and its constituents on fetal development as well as indirect effects, namely placental insufficiency, on postnatal metabolic diseases.

## 1. Introduction

The worldwide use of cannabis has increased over the past decades [1], and among pregnant women, the usage can be as high as 20% between the ages of 18–24 [2]. Moreover, in a separate study, it has been demonstrated that up to 35% of individuals had consumed cannabis at the time of a confirmed pregnancy [3]. Reports indicate that this is attributed to self-medication given the perception that cannabis is safe and can alleviate common pregnancy ailments including nausea, anxiety, and depression [4,5,6]. For example, in Colorado, ~70% of dispensaries recommended the use of cannabis to treat nausea associated with pregnancy [7]. A recent study has further shown that although the majority of mothers understood that the constituents of cannabis are transmitted to the fetus, some still decided to use it, which suggests that there is a misunderstanding regarding its safety in fetal life [8]. These misconceptions surrounding maternal cannabis usage warrant a closer look at the potential harms they could exert on the short- and long-term health of the baby.

Several clinical studies have linked prenatal cannabis exposure to placental abnormalities and fetal growth restriction [9,10,11,12]. However, while clinical studies link maternal cannabis use and low-birth-weight outcomes [9,10,11], many do not control for socioeconomic status and polydrug use. In addition, for ethical reasons, there are no randomized control trials that study the effects of maternal cannabis consumption on neonatal outcomes. Therefore, the safety of cannabis and its constituents needs to be addressed explicitly in animal models given the confounding issues of clinical studies. These constituents include Δ9-THC (the major psychoactive cannabinoid in cannabis) and cannabidiol (CBD, the largest non-intoxicating component of cannabis). To date, clinical studies indicate that prenatal exposure to cannabis can lead to FGR and neurological deficits [9,10,11,12,13,14]. In humans, intrauterine growth restriction (IUGR) often results due to placental insufficiency and is defined as the inability for the fetus to reach its full genetic growth potential [15,16]. It is well-known that these fetal growth deficits increase the risk of metabolic syndrome and cardiovascular disease in the offspring later in life [17,18,19,20,21]. IUGR can be followed by a period of accelerated growth, coined postnatal “catch-up growth”, which exacerbates the adverse metabolic consequences long-term [22]. These emerging links between Δ9-THC and FGR are of great concern considering that Δ9-THC has increased from 8.9% in 2008 to 17.1% in 2017 [23]. Moreover, Δ9-THC can readily cross the placental barrier and concentrate in fetal tissue [24,25]. Collectively, this suggests that maternal Δ9-THC exposure leading to FGR (Δ9-THC-induced FGR) could result in long-term dysmetabolism in the offspring. However, further research is warranted. Moreover, the effects of prenatal CBD exposure (in either the absence or presence of Δ9-THC) on maternal–fetal outcomes and postnatal health remain understudied despite CBD’s growing popularity and its perception as a “good” cannabinoid. Reports indicate that up to 62% of individuals use CBD to treat pain, anxiety, and depression [26], all common symptoms associated with pregnancy.

To appreciate how these constituents of cannabis might influence fetal development, it is first imperative to understand the endocannabinoid (eCB) system. The eCB system is composed of two main receptors, cannabinoid receptor type 1 (CB1) and type 2 (CB2), which are G protein-coupled receptors (GPCR) bound by endogenous eCB lipid ligands made up of polyunsaturated fatty acids found in the brain and peripheral organs [27]. The major endogenous agonists of the eCB system are a class of eicosanoid cannabinoids called 2-arachidonylglycerol (2-AG) and anandamide (AEA) [28,29]. Other classes of cannabinoid agonists include classical tricyclic dibenzopyrans (e.g., Δ9-THC, HU-210), bicyclic and tricyclic analogs of Δ9-THC (e.g., CP-55,940), and aminoalkylindole cannabinoids (e.g., WIN55212). Although CB1 and CB2 receptors recognize cannabinoid agonists with the same structural groups, they differ by their affinity in some cases [30]. It is thought that AEA is a high-affinity partial agonist of CB1 with low affinity and activity at CB2, while 2-AG is a moderate-affinity full agonist at CB1 and CB2 [30,31]. CB1 and CB2 generally interact with heterotrimeric G protein, Gα_i/o_, which inhibits adenylyl cyclase or couples with the mitogen-activated protein kinase. Just like other GPCRs, CB1 and CB2 have other effector proteins (e.g., β -arrestin-1), which likely allow them preferentially select toward a particular pathway depending on the type of ligand; this is called biased signaling [32].

The eCB system was originally thought to be localized primarily in the central nervous system (CNS) with its primary role in regulating neurotransmission. It is now well-established to be also present in peripheral tissues. The eCB system emerges early in development; not only are CB1 and CB2 expressed in embryonic development, but they also play a role in implantation and placentation, suggesting that disruption in this system could lead to adverse outcomes in pregnancy [33,34]. However, to date, the majority of preclinical and clinical studies focus on examining perinatal cannabinoid exposure on the neurodevelopmental and behavioral outcomes of the offspring [35,36,37,38,39,40,41] while not addressing the postnatal cardiovascular and metabolic outcomes that might be involved. Notably, there is evidence that both CB1 and CB2 receptors are found in peripheral fetal/postnatal tissues (i.e., heart, liver, adipose, pancreas) [34,42,43,44,45,46], which supports the notion that cannabinoids could directly impact fetal and postnatal development. In addition, there is a decent body of knowledge regarding the eCB system and its role in metabolic diseases (as reviewed in [47]). Specifically, the eCB system plays a role in food intake, energy expenditure, lipid metabolism, insulin sensitivity, and cardiovascular disease [47]. This could further suggest that developmental abnormalities in the development of the eCB system itself could lead to metabolic disease later in life. Given what is known to date about the effects of Δ9-THC, we postulate that cannabinoids can negatively impact placental function and lead to indirect effects (e.g., placental insufficiency) on the long-term health on the offspring. In addition to this, we speculate the possibility that major lipophilic constituents, namely, Δ9-THC and/or CBD, could also have direct effects, by which they cross the placenta and influence fetal organ development via the endocannabinoid (eCB) system. Therefore, in this review, we aim to highlight the current knowledge of the effects of cannabis and its constituents, on the placenta and postnatal metabolic health of the offspring, with an emphasis on Δ9-THC.

## 2. The Impact of Cannabinoids on the Placenta 

IUGR is the main adverse outcome of placental insufficiency [15,16]. Since gestational use of cannabis has been associated with FGR [9,10,11,12], it is therefore critical to address whether the placenta is a root cause of cannabinoid-induced FGR and the subsequent metabolic deficits in postnatal life.

### 2.1. The Endocannabinoid System in Placental Development and the Influence of Exogenous Cannabinoids 

The two major eCBs, 2-AG and AEA, and the main eCB receptors are found in rodent placenta [33]. Both fatty acid amide hydrolase (FAAH) and N-acylphosphatidylethanolamine-specific phospholipase D (NAPE-PLD) are responsible for metabolizing and synthesizing AEA, respectively, and can be found in both the human and rodent placenta [33]. It is important to note that data on 2-AG and its role throughout gestation remain elusive. The eCB system is present in the midgestational rodent placenta, and research suggests that endocannabinoids, mainly AEA coupled with the activity/expression of its corresponding receptors and enzymes, play a vital role in decidualization, placentation, and the maintenance of pregnancy [33,48]. With respect to the maternal side, CB1, CB2, NAPE-PLD, and FAAH are expressed in the decidua of both human and rodents and are thought to also play a role in decidualization, placental development, and maintenance of pregnancy [49,50,51,52]. When endometrial stromal cells decidualize, they naturally increase the transcript abundance of CB1 and CB2 receptors [53], which could make decidualized cells particularly sensitive to sustained action by eCBs and exogenous cannabinoids (i.e., naturally occurring plant cannabinoids or synthetic cannabinoids), resulting in compromised decidual function [54]. Near the pinnacle of rodent decidual development, NAPE-PLD increases along with AEA [55,56]; however, this is followed by an increase in the AEA-degrading enzyme, FAAH [57], indicating that there are changes in the eCB profile during gestation. After the peak of decidualization, the eCB system continues to play a major role in placentation. For example, CB1 knockout mice exhibit hampered trophoblast proliferation and invasion along with spongiotrophoblast development [48], which could ultimately lead to FGR. In human BeWo cells, increases in AEA impair trophoblast proliferation and induce apoptosis [58,59], while an increase in 2-AG also leads to apoptosis via the CB2 receptor [60]. Moreover, aberrant expression of placental NAPE-PLD, CB1, FAAH, and AEA in the first trimester of pregnancy results in adverse pregnancy outcomes such as spontaneous miscarriages [61,62,63]. In vitro, in vivo, and clinical studies implicate that increased levels of AEA in plasma are associated with pregnancy complications such as endometriosis and miscarriage; however, the contributions of 2-AG in early pregnancy outcomes remain understudied [64]. Notably, it is thought that too much or too little of AEA could both negatively impact placental development [50]. Collectively, it is apparent that a coordinated balance in the eCB system (e.g., ligands, receptors, and enzymatic profiles) is necessary for proper embryo implantation and placentation. 

It is conceivable that the introduction of exogenous cannabinoids such as Δ9-THC and/or CBD could influence the homeostasis of the eCB system during the implantation and development of the placenta. Indeed, in an ex vivo model of the human placenta, treatment with Δ9-THC altered the eCB system, whereby NAPE-PLD was initially increased at 24 h and FAAH exhibited an opposite effect [65]. This culminated in an increase in AEA 72 h post-treatment [65]. It has been previously shown that an increase in AEA disrupts the fine-tuned balance of apoptosis in cytotrophoblasts and impairs placental hormone synthesis [59,66]. Furthermore, in vitro models demonstrate that Δ9-THC can also disrupt trophoblast differentiation, proliferation, and syncitialization [53,67,68,69]; however, the body of knowledge with respect to CBD and other cannabinoids is limited. One study demonstrated that Δ9-THC, CBD, and/or Cannabinol (CBN) (0.5 μM) can suppress both endometrium stromal cell decidualization and trophoblast invasion, suggesting these cannabinoids impair the communication between the endometrial and trophoblast cells [53]. In contrast, a recent study found that only CBD impairs decidualization in vitro [70]. This contradiction could be attributed to the concentration of Δ9-THC (10 μM) in the latter study and the fact that cannabinoids can have the potential for a dose-dependent dual response [53]. One proposed mechanism by which CBD impairs decidualization is through increases in AEA levels, which are implicated to impair decidualization [71]. This mechanism is further supported by the fact that CBD can prevent degradation of AEA by inhibiting FAAH in mice [72]. Given this, and the role the eCB system plays in early gestation, it is apparent that exposure to some exogenous cannabinoids during gestation has the potential to disrupt the function of the eCB system during implantation and placentatal development, adversely impacting pregnancy outcomes and fetal development.

### 2.2. Prenatal Exogenous Cannabinoid Exposure on Placental Insufficiency and Birth Outcomes 

Systemic reviews and meta-analyses suggest that maternal cannabis consumption is associated with poor birth outcomes including, but not limited to, pre-term deliveries, increased neonatal intensive care unit admission, and low birthweight [9,10,11,73,74,75]. However, many of these studies do not account for the frequency of usage, concentrations of Δ9-THC, and/or CBD and are confounded by socioeconomic status and polydrug use. Therefore, it is imperative to employ animal models to explicitly address the contributions of cannabis and its individual constituents (i.e., Δ9-THC and CBD) to maternal–fetal outcomes while controlling for environmental factors.

In animal models, prenatal studies using doses of around 3 mg/kg of Δ9-THC (i.p.) result in rodent plasma concentrations (8.6–12.4 ng/mL) similar to those of cannabis users (13–63 ng/mL), 0–22 h post inhalation from a 7% Δ9-THC content joint [76,77]. A similar concentration range was also reported in aborted human fetal tissue and placentae from cannabis users [78]. For CBD, established methods using meconium and umbilical cord samples from newborns for the detection of in utero cannabinoid exposure demonstrated that the range of CBD varies from 10–335 ng/mL [79]. In pregnant mice, 10 mg/kg (via tail vein injection) results in peak maternal serum concentrations of 2615.3 ± 442.3 ng/mL and peak fetal tissue concentrations of 598.7 ± 251.9 ng/g of fetus (whole body measurement) [80].

Recent studies using 3 to 5 mg/kg Δ9-THC (i.p.) during gestation have reported placental abnormalities and fetal growth deficits [67,81]. However, the study by Chang et al. demonstrated that exposure to 5 mg/kg Δ9-THC (i.p.) in mice led to fetal demise and decreases in litter size [67]. This was attributed with lower expression of placental CB1 and CB2 [67], which could suggest disrupted endocannabinoid signaling in the placenta. A follow-up study also found impaired placental angiogenesis in 5 mg/kg Δ9-THC-exposed mice, and it also reported that placentae from women who smoked cannabis (no alcohol or tobacco) during pregnancy had decreased blood vessel formation (low-CD31-integrated optical density) with narrowed placental blood vessels [82]. Interestingly, in the same study, Δ9-THC in vitro reduced cell migration in human umbilical vein endothelial cells (HUVEC), which was partially reversed by CB1 and CB2 antagonists [82]. However, it is important to note that this 5 mg/kg dose of Δ9-THC led to fetal demise, which in itself could be a confounding variable in the interpretation FGR outcomes (e.g., litter size effect). In addition, this higher dose of Δ9-THC could also confound birth outcomes by influencing maternal behavior and physical measures (i.e., maternal weight gain), but these parameters were not assessed [83]. 

Clinical studies further support the notion that cannabis impairs fetal–maternal blood flow, as demonstrated in studies that found increased placental vascular resistance in mothers who consumed cannabis in pregnancy [84]. Recently, our laboratory group demonstrated that daily exposure to 3 mg/kg Δ9-THC (i.p.) in rats resulted in placental insufficiency (e.g., larger placenta) and symmetrical FGR [81]. The placental insufficiency was mediated by a lower fetal to placental weight ratio, diminished differentiated trophoblasts (e.g., lower epithelial cell adhesion molecule (EPCAM) in the labyrinth zone), decreased fetal blood space, and reduced labyrinth-specific expression in glucocorticoid receptor (GR) and glucose transporter 1 (Glut1) [81]. A larger placenta and a decreased fetal:placental weight ratio has also been reported in mice that inhaled 200 mg of cannabis smoke [85]. However, unlike previous studies, our lower dose of Δ9-THC did not impact litter size/fetal demise, maternal food intake, or maternal weight gain. These maternal outcomes are also supported by another group using a similar dose of Δ9-THC [83]. The increase in placental weight leading to placental insufficiency compliments human studies whereby cannabis use in pregnancy was associated with a larger placenta [12,86]. In human placental BeWo cells, it was further demonstrated that Δ9-THC, and not the inactive metabolite (e.g., carboxylate Δ9-THC), directly impairs placental GR and Glut1 [81].

With respect to CBD, the data available on placental physiology are extremely limited. However, similar to the previous report described earlier with Δ9-THC [82], it has been demonstrated that CBD impairs migration, invasion, and sprouting in HUVEC cells lines and angiogenesis in mice [87]. Feinshtein et al. further presents an interesting perspective on the impact of CBD on the placenta that broadens the potential indirect effects of cannabinoid exposure [88]. This ex vivo study found that high doses of CBD can inhibit breast-cancer-resistant protein (BCRP) [88], a multi-drug-resistant protein found in the syncytiotrophoblast that can remove a variety of compounds out of the cell, thereby potentially impairing the ability for the placenta to clear xenobiotics [89,90]. Subsequent studies by the same group also found that the function and expression of P-glycoprotein (P-gp), which is another placental gatekeeper protein, was also decreased by exposure to CBD [91]. Collectively, these studies suggests that exposure to CBD may lead to inadvertent downstream insults secondary to CBD exposure, whereby the protective role of the placental barrier is compromised, leading to an even greater risk of impaired fetal development.

In summary, these studies to date would seem to indicate that Δ9-THC alone leads to fetal growth deficits via alterations in placental perfusion, ranging from narrow maternal sinusoids to decreased angiogenesis and fetal blood space. Moreover, results from Natale et al. reveal that Δ9-THC impairs the placental expression of Glut1 in both the human and the rat, uncovering an additional mechanism for the fetal growth restriction observed [81]. Further studies are warranted to elucidate the underlying molecular mechanisms involved and to delineate the contributions of CBD alone (or in combination with Δ9-THC) on placental function and maternal–fetal outcomes. 

### 2.3. Underlying Mechanisms: Cannabinoids and Placental Subcellular Stress 

One underlying mechanism for cannabinoid-induced placental insufficiency may be subcellular stress. Lojpur and colleagues demonstrated that treatment with Δ9-THC to human undifferentiated cytotrophoblast BeWo cells induced endoplasmic reticulum (ER) stress (i.e., higher CHOP, ATF4, ATF6, spliced Xbp1) in a dose-dependent manner mediated by the CB1 and CB2 receptors [92]. This is interesting given idiopathic IUGR placentas exhibit ER stress [93] and pharmacological activation of ER stress (e.g., tunicamycin) leads to FGR through decreases in placental vascular endothelial growth factor 1 (VEGFR-1), placental glycogen content, and glucose transporter 1 [94]. Given exposure to Δ9-THC in rat pregnancy results in decreased placental Glut1 [81], chronic ER stress may be one of the mechanisms of Δ9-THC-induced placental insufficiency. In addition, Δ9-THC also induces mitochondrial dysfunction in undifferentiated and differentiated BeWo cells [69,92]. Δ9-THC also decreased mitochondrial size, impaired ATP production, and reduced mitochondrial respiration in vitro [92]. Many of these findings were reciprocated in a study by Walker et al., 2020, in differentiated cytotrophoblasts in association with oxidative stress (e.g., elevated SOD1 and SOD2) and impaired syncitialization [69]. As the mitochondria and ER are physically connected at sites called the mitochondrial-associated ER membrane (MAM), which can indirectly influence the production of ATP and respond to ER signaling, it is not surprising that placental oxidative stress also occurs in conjunction with ER stress [95]. Furthermore, these in vitro findings with Δ9-THC are consistent with studies that report mitochondrial dysfunction in IUGR placentas [93,96]. Overall, it seems that Δ9-THC-induced ER stress and mitochondrial dysfunction in placenta are consistent with other models of placental-insufficiency-induced IUGR. 

## 3. Cannabinoid-Induced FGR and Postnatal Hepatic Function and Lipid Metabolism

The liver has an essential role in managing lipid metabolism, and aberrant hepatic function can result in dyslipidemia, which is associated with obesity and impaired glucose tolerance [97]. It is well-documented by epidemiological studies that low-birth-weight outcomes lead to obesity and non-alcoholic fatty liver disease (NAFLD) later in life [20,98,99,100,101]. In fact, pre-clinical studies utilizing different means of in utero insults including nicotine-induced and nutrient restriction-induced FGR result in higher hepatic triglycerides and cholesterol, respectively [102,103]. We have recently demonstrated that maternal exposure to Δ9-THC i.p. in rats leads to liver growth deficits at birth followed by complete catch-up growth by 3 weeks whereby exposed offspring caught-up in body and liver weight relative to control [81]. In a follow-up study, Oke et al. demonstrated for the first time that Δ9-THC-induced FGR led to male-specific augmentation of hepatic lipid synthesis (e.g., DGAT2, ACCa, FABP1, and SCD) as early as 3 weeks [104]. This was partially sustained (DGAT2) in adulthood, culminating to increased hepatic triglycerides and visceral adiposity [104]. These metabolic deficits observed in adult rats (6 months) were associated with increased expression of p66shc [104], which targets the mitochondria and leads to pro-apoptotic reactive oxygen species (ROS) release [105], as well as induces intracellular lipid accumulation [106]. These mitochondrial defects are believed to be potentiated by catch-up growth [107], which is known to further increase the long-term risk of metabolic disease [108,109]. Another mechanism for the increase in hepatic ROS production in these Δ9-THC exposed rat offspring could be attributed to cannabinoid receptors associated with mitochondrial membranes (mtCB_1_) [110]. In astrocytes, activation of mtCB_1_ leads to decrease in mitochondrial complex I, leading to a reduction of ROS, alterations in the redox state, and ultimately impacting behavioral outcomes [110]. While the expression of mtCB_1_ has not be examined in peripheral tissues, it is tempting to speculate that if there is a loss of mtCB_1_ signaling by exogenous cannabinoids after birth, this could further contribute to the observed increase in complex I and ROS in the livers of Δ9-THC exposed rat offspring [104]. 

Oke et al. further elucidated that higher hepatic expression of p66shc in Δ9-THC-exposed offspring might also be regulated via epigenetic mechanisms. Δ9-THC offspring exhibited decreased expression of the hepatic microRNAs, miR-203a-3p and miR-29a/b/c [104]. Collectively, these microRNAs can influence the expression of p66Shc and long-term liver health [111,112,113,114]. This indicates that there are hepatic epigenetic modifications associated with an increase in hepatic triglycerides and mitochondrial dysfunction due to prenatal Δ9-THC exposure [104]. Overall, this study offers an early insight into how Δ9-THC-induced FGR leads to long-term dyslipidemia while highlighting the importance of catch-up growth as a major driver of these deficits. It also reveals that both mitochondrial and epigenetic mechanisms may underlie the dysmetabolism observed. However, to date, there are no studies that examine the long-term metabolic effects of prenatal CBD exposure on postnatal lipid homeostasis. If prenatal CBD exposure similarly leads to placental insufficiency and FGR, dyslipidemia may also occur in postnatal life. 

It is important to highlight that there can be additional direct effects of Δ9-THC in fetal liver development that warrant further investigation. This is key for metabolic health as the rodent liver tissue undergoes major developmental milestones in mid-gestation and continues to develop in postnatal life [115,116], while human livers are terminally differentiated at birth. In addition, cannabinoid receptors are expressed early in the fetal rodent liver with the expression profile of CB1/CB2 changing throughout development, implicating a potential role in liver development [34]. 

A third mechanism for prenatal cannabinoid-induced dysmetabolism in the liver may be due to direct changes in the development of the eCB system itself. Metabolic disease is associated with an altered eCB system [47], so it is plausible that if in utero exposure to exogenous cannabinoids alters the long-term function of eCB system in the offspring, it could potentially lead to the development of metabolic diseases. In fact, an adverse in utero environment (i.e., a high-caloric diet) has been shown to impact the endocannabinoid system in the postnatal liver and adipose tissue [42]. Unfortunately, there are currently no studies that address the impacts of prenatal cannabinoid exposure on the eCB system (i.e., CB1, CB2, AEA, 2-AG, and associated metabolic enzymes) homeostasis in postnatal life. 

Endocannabinoids, and particularly CB1, have been shown to play an important role in metabolic disease such as fatty liver disease, type II diabetes, diet-induced steatosis, and dyslipidemia and are generally associated with overactivity [117,118,119,120,121,122]. CB2 receptors could also be involved as an increase in CB2 receptors in NAFLD was found despite its lack of expression in a normal adult liver [34,123]. Mechanistically, agonism of the CB1 receptor increases fatty acid synthesis in the liver and leads to obesity in mice [122], and this was further supported by subsequent studies whereby CB1 knockout mice exhibited decreased lipogenesis in the liver, a decrease in diet-induced obesity, and increased leanness [121,124]. It is tempting to speculate that perhaps there might be epigenetic modifications in the eCB system due to prenatal cannabis exposure, which could lead to long-term or delayed alteration in the eCB system leading to the development of adult-onset metabolic diseases. This is conceivable because it has been highlighted that components of the eCB system (e.g., CB1, CB2, and FAAH) can be regulated by epigenetic mechanisms in neurodevelopment upon exposure to exogenous cannabinoids, as reviewed in [125]. While most studies to date only examine neurological mechanisms, it is possible that peripheral tissue could also exhibit epigenetic mechanisms.

## 4. Cannabinoid-Induced FGR and Postnatal Glucose Homeostasis 

The pancreas has a major role in glucose homeostasis. As previously mentioned, epidemiological studies indicate that FGR leads to metabolic syndrome, including T2D diabetes [21]. This is thought to be attributed to a “thrifty phenotype” whereby in utero deprivation leads to an adaptive energy-conserving phenotype [21]. In rodents, it has been previously demonstrated that prenatal exposure to environmental insults such as drugs (e.g., nicotine and SSRI) and protein restriction leads to impaired pancreatic function in fetal and postnatal life [126,127,128]. Therefore, cannabinoid-exposed offspring might be susceptible given that clinical studies suggest that cannabis leads to low-birthweight babies and placental abnormalities [9,10,11,12]. Moreover, we and others have demonstrated that specifically Δ9-THC leads to placental insufficiency and FGR [67,81]. Secondly, both the human and rodent adult and rodent fetal pancreas express components of the eCB system [43,129,130]. Despite this, there are still extremely limited preclinical studies, and no clinical studies, on the effects of prenatal cannabinoid exposure and the effects on postnatal pancreatic function and glucose homeostasis.

**Figure 1:**
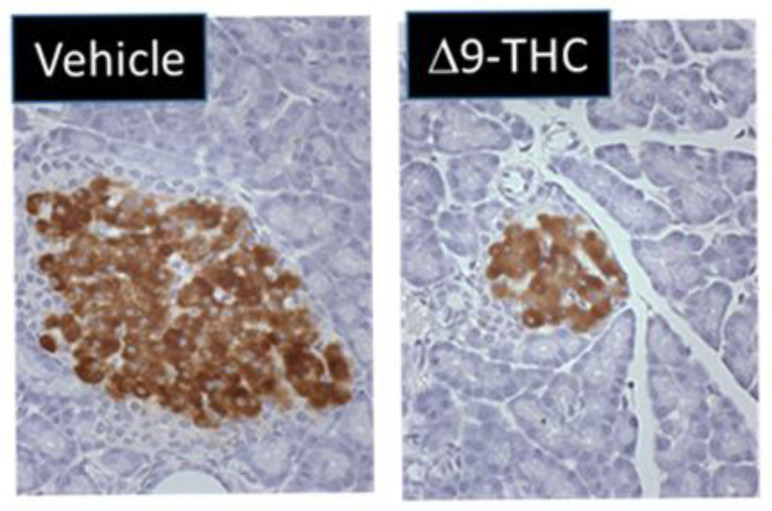
Maternal exposure to 3 mg/kg THC i.p. daily from gestational day 6–22 leads to reduced pancreatic islet size at 5 months in female offspring. Representative immunohistochemistry of pancreatic islets immunostained for insulin from vehicle and THC rat offspring.

One study by Gillies et al. demonstrated that gestational exposure to Δ9-THC leads to smaller pancreas-to-body-weight ratios at birth [131]. Interestingly, at 3 weeks of age, female Δ9-THC offspring exclusively exhibited decreases in both total and small-islet density along with a decrease in β-cell mass (41%) (Figure 1) [131] but no change in α cell mass [131]. This was associated with rapid postnatal catch-up growth. At 5 months, the observed sex-specific deficits persisted, and when challenged with a glucose tolerance test, female Δ9-THC-exposed offspring demonstrated glucose intolerance, despite an augmented insulin response [131]. Notably, these sex-specific effects were not associated with changes in sex steroid hormones (e.g., testosterone, estrogen, and progesterone) [131,132]. To address if peripheral insulin resistance might be occurring, Δ9-THC female offspring were challenged with insulin, and an impairment of insulin receptor function was observed (as indicated by a decrease in phosphorylated Akt [Ser 473]) in the soleus muscle [131]. This would suggest insulin receptor insensitivity [133]. It should be noted that fasting serum glucose and insulin levels remained stable, which implies that there might be some β-cell compensation that over time could lead to β-cell exhaustion, β-cell death, and ultimately the progression of T2D [134,135]. Overall, these Δ9-THC-exposed female offspring exhibit phenotypes resembling the progression to T2D. Further studies are warranted to address the effects of other constituents of cannabis (e.g., CBD) and the long-term impact on glucose homeostasis (e.g., >6 months).

Notably, despite the observed decrease in islet β cells, Gilles and colleagues did not observe any changes in proliferation and apoptosis in the pancreas at postnatal day (PND) 21 [131]. One possibility is that these deficits occurred early in gestation by direct effects of Δ9-THC on the pancreas. This is supported by the fact that the eCB system is present in fetal pancreatic tissue, and fetal endocannabinoids can directly affect proliferation and cellular organization of pancreatic islet cells [43]. Previous studies have indicated that CB1 activation in the pancreas can impair β-cell growth by cell cycle arrest and decrease survival by stimulating apoptosis [136]. However, to date, the direct sex-specific effects of Δ9-THC on fetal β-cell development remain elusive. Of course, the indirect effects of prenatal Δ9-THC on placental insufficiency and fetal organ deficits [67,81] must also be taken into consideration with respect to long-term glucose intolerance. 

As previously speculated in the liver, prenatal cannabinoids could also impact pancreatic function through developmental alterations in the eCB system. Preclinical studies have demonstrated that activation of CB1 receptors promotes lipogenesis and drives diet-induced obesity [122], whereas CB1 knockouts not only showed opposite effects but also exhibited no signs of associated insulin resistance [121,124]. Moreover, diabetic patients exhibit higher serum concentrations of AEA and 2-AG during hyperglycemia [137]. It seems apparent that the overactivation of the eCB system is associated with T2D. One study delineated the involvement of eCB system with respect to β-cell loss in T2D [138]. Specifically, in vivo CB1 blockade and macrophage CB1 knockouts were demonstrated to prevent macrophage infiltration in islet cells, which alleviated T2D phenotypes such as hyperglycemia and impaired glucose-induced insulin secretion [138]. Interestingly, clinical trials support the benefits of CB1 antagonists, namely rimonabant, in T2D patients, as they led to improvements in glycemic control [139,140]; however, rimonabant was withdrawn from the market due to its serious psychological side-effects. Given the aforementioned glycemic impairments in glucose homeostasis in Δ9-THC offspring [131], along the eCB systems involvement in pancreatic development [43], there might be a possibility that in utero cannabinoid exposure leads to long-term alterations in the eCB system; however, more studies need to be conducted.

## 5. Cannabinoid-Induced FGR and Postnatal Cardiovascular Function

**Figure 2:**
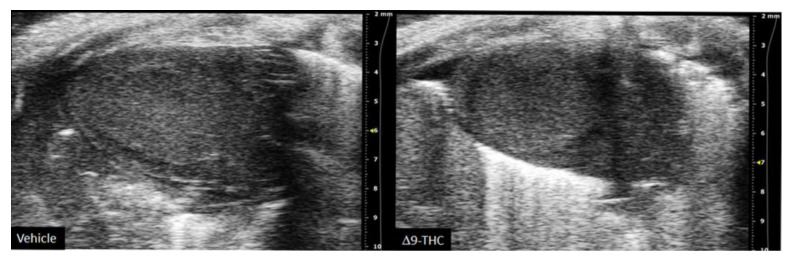
Maternal exposure to 3 mg/kg i.p. daily from gestational day 6–22 results in decreased heart size and stroke volume at postnatal day 1. Representative echocardiography (long-axial view of the left ventricle) from vehicle and THC rat offspring at postnatal day 1.

There are currently limited studies that link cannabis use in pregnancy to long-term cardiovascular dysfunction. This is crucial given prenatal cannabis or Δ9-THC alone results in FGR [9,10,11,12,67,81] and that some of the earliest work in the field of developmental origins of health and disease (DOHaD) associated low-birthweight outcomes to an increased risk of cardiovascular disease [17,18]. Furthermore, preclinical animal studies have demonstrated the adverse effects of maternal protein restriction, nicotine and hypoxia on the long-term cardiac outcomes in offspring [141,142,143,144]; however, no studies have made the link between maternal cannabinoid exposure to long-term cardiac deficits. While clinical studies demonstrate that adult cannabis use leads to adverse cardiovascular outcomes [145,146], the long-term effects of prenatal exposure remains elusive. A recent retrospective cohort study did speculate that cannabis exposure in utero might be detrimental to heart development given babies born to cannabis had an increased risk of death within 1 year of birth [OR = 1.35, 95% CI = 1.12, 1.62] [147]. However, the definitive effects of constituents of cannabis (i.e., Δ9-THC, CBD) on the developing heart warrant further investigation. Recently, we demonstrated that rat offspring exposed to Δ9-THC in utero resulted in cardiac growth deficits at birth (Figure 2) [148], followed by cardiac remodeling and impaired cardiac function at 3 weeks [148]. Specifically, at 3 weeks of age, Δ9-THC-exposed pups exhibit complete catch-up growth concomitant with early signs of ventricular hypertrophy and increased fibrotic markers, namely collagen type 1 and 3 [148]. This was associated with lower stroke volume and cardiac output. These elevated markers of cardiac hypertrophy and fibrosis have been previously demonstrated in IUGR models of maternal nicotine, hypoxia, and protein restriction [141,142,144]. While these aforementioned studies observed these effects around adulthood (3–7 months old), it is noteworthy that the Δ9-THC offspring began to exhibit deficits at 3 weeks [148]. Unfortunately, our study did not examine effects in the females, and further studies should investigate whether these cardiac deficits persist later in life. 

It is reasonable to propose that direct and/or indirect effects might adversely impact the developing heart. As previously stated, indirect effects include placental deficiency leading to FGR, which increases the risk of cardiovascular disease in adulthood [17,18]. Furthermore, subsequent postnatal catch-up growth in these Δ9-THC offspring may exacerbate the cardiac defects already observed at birth. In fact, early clinical studies found that low-birthweight females with the highest body weight by 1 year old were at greater risk of coronary heart disease in adulthood [19]. This provides a foundation that postnatal catch-up growth can negatively impact the heart. Direct effects could also be at play during fetal development given that cannabinoids can cross the placenta and Δ9-THC has been demonstrated to inhibit cardiomyocyte growth in isolated rat cardiomyocytes [149]. Moreover, CB1 antagonists prevent Doxorubicin-(a chemotherapy medication)-induced apoptosis in embryonic heart cells [150], while CB1 agonists (AEA, Δ9-THC, and HU-210) have been demonstrated to decrease mitochondria respiration and mitochondrial membrane potential in rat heart mitochondria [151]. Overall, these data suggest that maternal exposure to Δ9-THC leads to early onset of cardiac dysfunction associated with postnatal catch-up growth. Whether these cardiac deficits worsen into adulthood remains to be determined, especially given the dyslipidemia and dysglycemia exhibited in these offspring [104,131].

Impaired eCB system development may further play a mechanistic role given both CB1 and CB2 are expressed in the heart, and the eCB system is involved in many cardiac pathologies [152,153,154]. In short, evidence suggests that CB2 activation seems to reduce infarct size in models of ischemic-reperfusion injury [155]. Secondly, CB1 antagonism improved doxorubicin-induced cytotoxicity, hepatic cirrhosis cardiomyopathy, and diabetes-associated cardiac fibrosis, as reviewed in [155]. Moreover, CB1 and CB2 exhibit opposing effects, with CB1 upregulation and CB2 downregulation being associated with inflammation in atherosclerosis [155]. Future studies should examine eCB alteration in long-term metabolic disease associated with prenatal-cannabinoid-induced FGR and whether there are long-lasting/delayed effects affecting eCB homeostasis in the heart.

## 6. Cannabinoid-Induced FGR and Postnatal Reproductive Function 

Cannabis has been shown to lead to low-birthweight outcomes [9,10,11], and preclinical studies using different maternal insults (i.e., nutrient restriction) indicate that FGR also negatively impacts offspring ovarian follicles [156,157,158]. This is not surprising since primordial follicle assembly occurs in utero and generally dictates the number of oocytes in the offspring. While cannabis consumption has been reported to adversely impact adult female fertility [33], the effects of gestational exposure on the offspring is not well-understood.

To date, very little is known regarding the effects of prenatal cannabinoids on reproductive organ development and function. While evidence in humans is lacking, an elegant study by Castel and colleagues demonstrated that prenatal synthetic CB1/2 agonists (0.5 mg/kg WIN55212) led to long-term decreases in ovarian reserve associated with altered eCB enzymatic expression in rodent offspring at 3 months [159]. These effects were shown to be mediated by CB1, as the introduction of an inverse agonist reversed these effects. Interestingly, these effects were not observed at earlier time points (PND 6 and PND 40), which suggests these effects were delayed [159]. While this study could suggest that fetal ovarian CB1 activation leads to deficits in ovarian reserve in adulthood, the underlying molecular mechanisms resulting in this delay is still unknown. One proposed mechanism was that there could be increased follicular atresia; however, this was not examined. Alternatively, given that this study observed long-term alterations in ovarian eCB enzymes, they also highlight how interruption of eCB in ovarian development could have lasting epigenetic modifications (as reviewed in [13]); therefore, more studies should examine the potential epigenetic modification in exposed offspring ovarian follicles. Notably, this group did not report any data on birth outcomes and did not observe any deficits in ovarian reserve in 3-month Δ9-THC-exposed offspring. Building on this, Martinez-Pẽna et al. recently demonstrated that 3 mg/kg of Δ9-THC during gestation led to accelerated folliculogenesis and follicular arrest in 6-month-old offspring underpinned by reduced blood vessel density along with a decrease in pro-angiogenic (i.e., VEGF and VEGFR-2) and increase in anti-angiogenetic (i.e., TSP-1) markers. Although there was not any reported follicular atresia (e.g., apoptosis) observed (*p* = 0.197), it could very well progress later with age. Further studies are necessary to examine the impact of prenatal cannabinoids on fertility in females and upon testicular function and fertility in Δ9-THC-exposed male offspring. Interestingly, an early rodent study found that maternal exposure to CBD and CBN, but not Δ9-THC, led to male offspring with decreased spermatozoa by almost 20% and significantly fewer live progeny themselves [160]. There may also be epigenetic mechanisms involved given that Δ9-THC and cannabinoid use can influence sperm DNA methylation and histone modifications in both humans and rats [161]. 

## 7. Future Studies

### 7.1. Windows of Maternal Exposure

The studies mentioned in the current review primarily address gestational exposure to cannabinoids; however, lactational windows of exposure are also important windows of development for the postnatal liver, adipose, and pancreases [116,162,163,164]. In addition, although hearts are generally assumed to be terminally differentiated at birth, rodent hearts have been shown to undergo a developmental transition period 3–4 days after birth where they quickly shift from hyperplasia to hypertrophy [165]. This is well within the rodent breast-feeding period. It is likely that if exposure to cannabinoids continue from gestation through lactation, the postnatal metabolic consequences would worsen. Moreover, further studies also need to address whether shorter exposure windows in gestation could decrease the impact on the placenta and developing fetus. Models with shorter windows of exposure early in gestation may mimic the tendency for pregnant women to consume cannabis only to alleviate the symptoms of morning sickness.

### 7.2. Intergenerational Effects of Maternal Cannabinoid Exposure

Epidemiological studies have demonstrated that the perinatal nutritional status of the mother can have effects on subsequent generations given that F2 offspring, born from F1 parents that were undernourished in utero, exhibit increased risk of metabolic disease (i.e., increased adiposity) later in life [166,167]. Animal studies also suggest that different maternal insults such as low-protein diet [168], undernutrition [169,170], and high-fat diet [171,172] lead to dysmetabolism across generations. Given maternal Δ9-THC exposure leads to FGR and dysmetabolism in FI offspring [81,104,131], it is imperative to address whether the F1 offspring metabolic deficits persists across progeny. In addition, as previously mentioned, studies with mice found that maternal exposure to cannabinoids significantly decreased male offspring fertility [160]; they also adversely affected female offspring gonads [132,159]. Collectively, this suggests that prenatal cannabinoids could impact both male and female gamete function, increasing the likelihood of intergenerational perturbations. 

### 7.3. Paternal Cannabinoid Exposure 

Current research in the field of DOHaD tends to focus solely on the maternal environment. It is important to address the paternal environment as it may also impact the development of the fetus. In fact, preclinical and epidemiological studies suggest that an adverse pre-conceptual paternal environment (i.e., obesity, diabetes, smoking, alcohol, and bisphenol A exposure) influences placental function and maternal–fetal outcomes which can lead to dysmetabolism in the offspring [173,174,175,176,177,178,179]. With respect to cannabinoid exposure, there is recent evidence that indicates that paternal cannabis and Δ9-THC exposure leads to DNA methylation in human and rat sperm, respectively [161], which suggests that it could have downstream consequences for the offspring. Indeed, offspring paternally exposed to Δ9-THC exhibited deficits in cholinergic synapse signaling and impaired cognitive function [180,181,182]. Recently, it was demonstrated that 1.5 mg/kg i.p. daily for five consecutive days of selective CB2 agonist (JWH-133) led to decreased testis size and sperm count in mice [183]. When exposed males were mated with untreated females, there was impaired placental development (decrease in spongiotrophoblast area and increased in labyrinth area) and reduced fetal weights (at e13.5 and e18.5) and birth weights [183]. However, to date, there are no studies that examine the effects of paternal exposure to Δ9-THC and/or CBD on the placental or metabolic health of the offspring. More studies are warranted to establish this causal link.

### 7.4. Other Receptors That Are Targeted by Cannabinoids

It is noteworthy that there are receptors other than the canonical cannabinoid receptors, CB1 and CB2, that cannabinoids can act upon. Namely, orphan G protein-coupled receptors 18 (GPR18), 55 (GPR55), and 119 (GPR119) can be activated by cannabinoids [184,185]. There are data that suggest GPR55s association with the eCB system, as it can heterodimerize with CB2 [186]. In addition, glycerophosphodiesterase 3 can metabolize GPR55 ligand, arachidonoyl lysophosphatidylinositol (LPI), into 2-AG and also act as a signaling switch between GPR55 and CB2, thereby linking GRP55 to canonical cannabinoid receptors [187,188]. Moreover, neurological studies indicate that exogenous cannabinoids (i.e., CBD) also act on other noncanonical cannabinoid-sensitive receptors, such as transient receptor potential vanilloid 1 and opioid receptor μ and δ [189], that may be of interest in peripheral tissues. With respect to metabolic syndrome, emerging data suggest that GPR55 and LPIs are associated with obesity and adipogenesis [190]. Furthermore, data indicate that GPR55 could influence insulin secretion and subcellular stress in in β-cells [191,192]. More recently, it was demonstrated that GPR55 may have a role in regulating cardiac function, cardiac immune homeostasis, and remodeling in mouse cardiomyocytes [193]. Given the potential role of these receptors in metabolic and cardiovascular pathophysiology, further investigation into these receptors is warranted.

## 8. Conclusions

**Figure 3:**
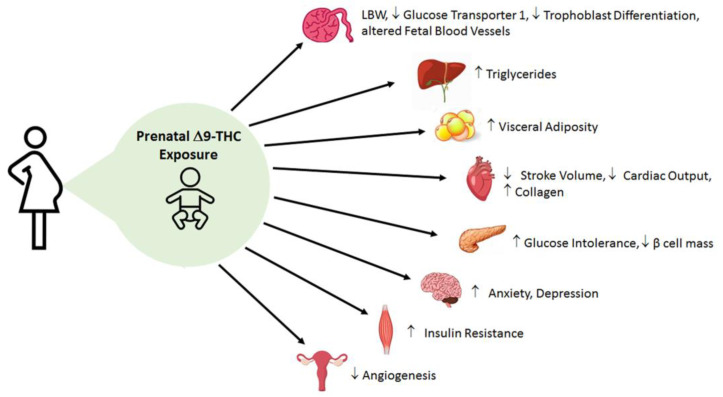
Overview of the postnatal metabolic outcomes of gestational exposure to THC. Maternal exposure to 3 mg/kg i.p. daily from gestational day 6–22 leads to postnatal alterations in the offspring’s placenta, heart, pancreas, liver, muscle, ovary, adipose, and brain.

Current preclinical data on the effects of perinatal cannabinoid exposure offer a captivating early insight into the metabolic consequences in the offspring. Although there are some mixed results in clinical data, pre-clinical animal studies support the idea that placental abnormalities and fetal growth deficits occur in cannabinoid-exposed offspring (Supplementary Table S1). This underscores the importance of utilizing an animal model to remove the confounding variables in clinical research (i.e., socioeconomic studies and polydrug use), as well as address the contributions of dosing, windows of exposure, and specific constituents of cannabis (i.e., Δ9-THC and CBD). Yet almost all animal studies primarily examine the effects of Δ9-THC alone. Nonetheless, emerging pre-clinical data suggest that Δ9-THC leads to placental insufficiency, early cardiac deficits, dysglycemia (i.e., glucose intolerance, blunted insulin signaling) and dyslipidemia (i.e., augmented hepatic triglycerides, and visceral adiposity) in adult offspring [81,82,104,131,148] (Figure 3). Furthermore, these data indicate that catchup growth may be playing a major role in exacerbating these observed deficits. Collectively, it seems that perinatal Δ9-THC exposure shares similar pathophysiology and patterns with FGR in general. However, it is also very likely that Δ9-THC and other cannabinoids also exhibit unique and direct effects as the eCB system is expressed in fetal tissues early in gestation and is involved in proper placentation and organ development [33,43]. Therefore, there is great impetus to better understand the mechanisms linking in utero cannabinoid exposure and its impact on fetal and postnatal development. Moreover, the role of the eCB system in metabolic diseases and its connection to prenatal cannabinoid exposure needs to be further investigated. These data are important as Δ9-THC content in cannabis is growing at a considerable rate [23,194]. In addition, CBD is extremely understudied with regards to its safety in pregnancy. This is still a vital question given that CBD is increasing in its popularity.

In summary, as it has been reported that almost 70% of pregnant and non-pregnant women perceive slight or no risk of harm in consuming cannabis once or twice a week [195], it is quite apparent that misconceptions still exist regarding its safety during pregnancy and postnatal life. Given this, along with increased popularity and legalization of cannabis, more studies are warranted to assess its safety to aid clinicians and policy-makers in evidence-informed decision-making. Moreover, with further understanding of the underlying mechanisms involved, safe interventions could be employed to ameliorate the detrimental metabolic outcomes for children who without choice were exposed to cannabinoids in utero.

## Supplementary Materials

The following are available online at https://www.mdpi.com/article/10.3390/ijms22179528/s1.

## Abbreviations

Δ9-THC	Δ9-tetrahydrocannabinol
2-AG	2-arachidonoylglycerol
AEA	anandamide
BCRP	breast cancer resistant protein
CB1	cannabinoid receptor type 1
CB2	cannabinoid receptor type 2
CBD	cannabidiol
CBN	cannabinol
CNS	central nervous system
DOHaD	developmental origins of health and disease
eCB	endocannabinoid
EPCAM	epithelial cell adhesion molecule
ER	endoplasmic reticulum
FAAH	fatty acid amide hydrolase
FGR	fetal growth restriction
GR	glucocorticoid receptor
Glut1	glucose transporter 1
GPCR	g protein-coupled receptor
GPR	orphan G protein-coupled receptor
HUVEC	human umbilical vein endothelial cells
IUGR	intrauterine growth restriction
MAM	mitochondrial-associated ER membrane
mtCB_1_	mitochondrial cannabinoid receptor type 1
NAFLD	non-alcoholic liver disease
NAPE-PLD	N-acylphosphatidylethanolamine-specific phospholipase D
P-gp	P-glycoprotein
ROS	reactive oxygen species
T2D	type II diabetes
VEGFR-1	vascular endothelial growth factor 1
