# Supplementary material for: Metabolic Consequences of Gestational Cannabinoid Exposure
Source: Int J Mol Sci. 2021 Sep 2;22(17):9528. doi: 10.3390/ijms22179528 (PMC8430813; doi:10.3390/ijms22179528)
Supplement: Supplementary file 1 [file ijms-22-09528-s001.zip › ijms-1357879-supplementary.pdf]

**Table S1** – Summary of Key Clinical and Preclinical Outcomes Regarding the Effects of Gestational Cannabis Exposure on Birth, Placental and Metabolic Outcomes

| Study | Model/Organism | Drug and/or Dosage | Birth Outcomes | Placental Outcomes | Offspring Metabolic Outcomes |
|---|---|---|---|---|---|
| **(English et al., 1997)** | Clinical | Cannabis | ↓ BW | N/A | N/A |
| **(Gunn et al., 2016)** | Clinical | Cannabis | ↓ BW<br>↑ NICU admission | N/A | N/A |
| **(Conner et al., 2016)** | Clinical | Cannabis | ↓ BW, except when controlled for tobacco use<br>↑ Preterm delivery, except when controlled for tobacco use | N/A | N/A |
| **(Carter et al., 2016)** | Clinical | Cannabis | N/A | ↑ Placental weight | N/A |
| **(Bailey et al., 2020)** | Clinical | Cannabis | ↓ BW<br>↑NICU admission<br>↑ Preterm delivery<br>↓ Apgar score | N/A | N/A |
| **(Singh et al., 2020)** | Clinical | Cannabis | ↓ BW<br>↑ NICU Admission | N/A | N/A |

| | | | | | |
|---|---|---|---|---|---|
| | | | ↑ pre-term delivery | | |
| **(Brar et al., 2019)** | Clinical | Cannabis | ↓ BW | ↑ Placental vascular resistance | N/A |
| **(Maia et al., 2019)** | Preclinical (*ex vivo* w/ human placentae) | Δ9-THC (10-40 μM) | N/A | ↑ Placental NAPE-PLD<br>↓ Placental FAAH<br>↑ Placental AEA | N/A |
| **(Neradugomma et al., 2019)** | Preclinical (*in vitro* w/ THESC) | Δ9-THC, CBD, CBN (0.2, 2 and 20 μM) | N/A | ↓ Decidualization<br>↓Trophoblast-endometrial stromal cell interactions<br>↓ Trophoblast invasion | N/A |
| **(Chang et al., 2017)** | Preclinical (*in vitro* w/ BeWo cells, *in vivo* w/ mice and human placenta) | Δ9-THC (0.3 to 30μM) *in vitro;* Δ9-THC (5 mg/kg *i.p.* GD5.5-18.5) *in vivo*; human cannabis smoker controlled for tobacco and alcohol | ↓ BW<br>↓ Litter size | ↓ Trophoblast migration and invasion *in vitro*<br>↓CB1 and CB2 in humans and animals | N/A |
| **(Khare et al., 2006)** | Preclinical (*in vitro)* | Δ9-THC (15 μM) | N/A | ↓Trophoblast proliferation | N/A |
| **(Walker et al., 2020)** | Preclinical (*in vitro)* | Δ9-THC (20 μM) | N/A | ↓ Syncitialization<br>↑ Oxidative stress | N/A |

| | | | | | |
|---|---|---|---|---|---|
| | | | | ↓ Fetal growth hormones<br>↓ Mitochondrial respiration | |
| **(Almada et al., 2020)** | Preclinical (*in vitro* w/ THESC (St-T1b) cells) | Δ9-THC (10 μM) and/or CBD (2 μM) | N/A | ↓ Decidualization by CBD but not Δ9-THC<br>↓Differentiation by CBD but not Δ9-THC<br>↓ mRNA levels of CYP19A1 and estradiol by CBD but not Δ9-THC | N/A |
| **(Chang et al., 2018)** | Preclinical (*in vitro* w/ HUVECs; *in vivo* w/ mice and human placenta | Δ9-THC *in vitro* (0–20 μM); Δ9-THC (5 mg/kg *i.p.* GD5.5-18.5)*in vivo* w/ mice; Cannabis inhalation in humans controlled for alcohol and tobacco | N/A | ↓ HUVEC migration and tube formation<br>↓Placental angiogenesis in mice<br>↓Placental angiogenesis in humans | N/A |
| **(Natale et al., 2020)** | Preclinical (*in vivo* w/ rats and *in vitro* w/ BeWo cells) | Δ9-THC *in vivo* (3 mg/kg *i.p.* from GD6-22) and *in vitro* (15 μM) | ↓ BW<br>No changes in litter size, maternal | ↑ Placental weight<br>↓Fetal:placental weight ratio<br>↑Placental | N/A |

| | | | | | |
|---|---|---|---|---|---|
| | | | weight gain and food intake<br>↓ Liver:body weight<br>↓ Brain:body weight | labyrinth area<br>↓ EPCAM expression<br>↓ Fetal blood space<br>↑ Pericytes recruitment<br>↓ Glut1 & GR | |
| **(Benevenuto et al., 2017)** | Preclinical (*in vivo* w/ mice) | 200 mg of Cannabis sativa 5 min of inhalation daily from GD5.5–GD17.5 | ↓ BW | ↑ Placental weight<br>↓ Fetal:placental weight ratio | N/A |
| **(Lojpur et al., 2019)** | Preclinical (*in vitro* w/ BeWo cells) | Δ9-THC (3 -30 μM) | N/A | ↑ ER stress via CB1 and CB2<br>↓Mitochondria respiration | N/A |
| **(Oke et al., 2021)** | Preclinical (*in vivo* w/ rats) | Δ9-THC (3 mg/kg *i.p.* daily from GD5.5-22) | ↓ BW<br>↓ Liver:body weight | N/A | ↑ Catch-up growth at PND21<br>↑ Markers of hepatic lipid synthesis at PND21 and 6 months<br>↑ Hepatic triglycerides at 6 months |

| | | | | | |
|---|---|---|---|---|---|
| | | | | | ↑ Hepatic mitochondrial dysfunction and oxidative stress<br>↑ Epigenetic (microRNA) markers associated with dyslipidemia<br>↑ Visceral adiposity |
| **(Gillies et al., 2020)** | Preclinical (*in vivo* w/ rats) | Δ9-THC (3 mg/kg *i.p.* daily from GD5.5-22) | ↓ BW<br>↓ Pancreas: body weight<br>↓ β-cell mass | N/A | ↓ Pancreatic total and small islet density at PND21 & 5 months<br>↑ Catch-up growth at PND21<br>↑ Glucose intolerance at 5 months<br>↓ β-cell mass at 5 months<br>↓ Peripheral insulin signalling |
| **(Lee et al., 2021)** | Preclinical (*in vivo* w/ rats) | Δ9-THC (3 mg/kg *i.p.* daily from GD5.5-22) | ↓ BW<br>↓ Heart: body weight<br>↓ Stroke volume<br>↓ Heart rate | | ↑ Catch-up growth at PND21<br>↓ Stroke volume<br>↓ Cardiac output<br>↑ Left ventricular wall thickness |

| | |
|---|---|
| | ↑ Cardiac protein expression of collagen type I & 3 |

Summary of key clinical and preclinical studies in this present review as it pertains to maternal cannabinoid exposure and its effects on the placenta, neonatal and postnatal metabolic outcomes. This table primarily focuses on cannabis and its constituents with an emphasis on preclinical findings. Δ9–THC: Δ9– tetrahydrocannabinol; AEA: anandamide; BW: birth weight; CB1: cannabinoid receptor type 1; CB2: cannabinoid receptor type 2; CBD: cannabidiol; CBN: cannabinol; EPCAM: epithelial cell adhesion molecule; ER: endoplasmic reticulum; FAAH: fatty acid amide hydrolase; GD: gestational day; Glut1: glucose transporter 1; GR: glucocorticoid receptor; HUVEC: Human umbilical vein endothelial cells; NAPE-PLD: N-acylphosphatidylethanolamine-specific phospholipase D; PND: postnatal day; THESCs: telomerase-immortalized human endometrial stromal cell line

**Table References**


Almada, M., Amaral, C., Oliveira, A., Fernandes, P. A., Ramos, M. J., Fonseca, B. M., Correia-da-Silva, G., & Teixeira, N. (2020). Cannabidiol (CBD) but not tetrahydrocannabinol (THC) dysregulate in vitro decidualization of human endometrial stromal cells by disruption of estrogen signaling. *Reproductive Toxicology*, *93*, 75–82. https://doi.org/10.1016/j.reprotox.2020.01.003

Bailey, B. A., Wood, D. L., & Shah, D. (2020). Impact of pregnancy marijuana use on birth outcomes: Results from two matched population-based cohorts. *Journal of Perinatology: Official Journal of the California Perinatal Association*, *40*(10), 1477–1482. https://doi.org/10.1038/s41372-020-0643-z

Benevenuto, S. G., Domenico, M. D., Martins, M. A. G., Costa, N. S., de Souza, A. R. L., Costa, J. L., Tavares, M. F. M., Dolhnikoff, M., & Veras, M. M. (2017). Recreational use of marijuana during pregnancy and negative gestational and fetal outcomes: An experimental study in mice. *Toxicology*, *376*, 94–101. https://doi.org/10.1016/j.tox.2016.05.020

Brar, B. K., Patil, P. S., Jackson, D. N., Gardner, M. O., Alexander, J. M., & Doyle, N. M. (2019). Effect of intrauterine marijuana exposure on fetal growth patterns and placental vascular resistance. *The Journal of Maternal-Fetal & Neonatal Medicine: The Official Journal of the European Association of Perinatal Medicine, the Federation of Asia and Oceania Perinatal Societies, the International Society of Perinatal Obstetricians*, 1–5. https://doi.org/10.1080/14767058.2019.1683541

Carter, R. C., Wainwright, H., Molteno, C. D., Georgieff, M. K., Dodge, N. C., Warton, F., Meintjes, E. M., Jacobson, J. L., & Jacobson, S. W. (2016). Alcohol, Methamphetamine, and Marijuana Exposure Have Distinct Effects on the Human Placenta. *Alcoholism: Clinical and Experimental Research*, *40*(4), 753–764. https://doi.org/10.1111/acer.13022

Chang, X., Bian, Y., He, Q., Yao, J., Zhu, J., Wu, J., Wang, K., & Duan, T. (2017). Suppression of STAT3 Signaling by Δ9-Tetrahydrocannabinol (THC) Induces Trophoblast Dysfunction. *Cellular Physiology and Biochemistry: International Journal of Experimental Cellular Physiology, Biochemistry, and Pharmacology*, *42*(2), 537–550. https://doi.org/10.1159/000477603

Chang, X., Li, H., Li, Y., He, Q., Yao, J., Duan, T., & Wang, K. (2018). RhoA/MLC signaling pathway is involved in $\Delta^9$-tetrahydrocannabinol-impaired placental angiogenesis. *Toxicology Letters*, *285*, 148–155. https://doi.org/10.1016/j.toxlet.2017.12.031

Conner, S. N., Bedell, V., Lipsey, K., Macones, G. A., Cahill, A. G., & Tuuli, M. G. (2016). Maternal Marijuana Use and Adverse Neonatal Outcomes: A Systematic Review and Meta-analysis. *Obstetrics and Gynecology*, *128*(4), 713–723. https://doi.org/10.1097/AOG.0000000000001649

English, D. R., Hulse, G. K., Milne, E., Holman, C. D., & Bower, C. I. (1997). Maternal cannabis use and birth weight: A meta-analysis. *Addiction (Abingdon, England)*, *92*(11), 1553–1560.

Gillies, R., Lee, K., Vanin, S., Laviolette, S. R., Holloway, A. C., Arany, E., & Hardy, D. B. (2020). Maternal exposure to Δ9-tetrahydrocannabinol impairs female offspring glucose homeostasis and endocrine pancreatic development in the rat. *Reproductive Toxicology (Elmsford, N.Y.)*, *94*, 84–91. https://doi.org/10.1016/j.reprotox.2020.04.070

Gunn, J. K. L., Rosales, C. B., Center, K. E., Nuñez, A., Gibson, S. J., Christ, C., & Ehiri, J. E. (2016). Prenatal exposure to cannabis and maternal and child health outcomes: A systematic review and meta-analysis. *BMJ Open*, *6*(4), e009986. https://doi.org/10.1136/bmjopen-2015-009986

Khare, M., Taylor, A. H., Konje, J. C., & Bell, S. C. (2006). Δ9-Tetrahydrocannabinol inhibits cytotrophoblast cell proliferation and modulates gene transcription. *Molecular Human Reproduction*, *12*(5), 321–333. https://doi.org/10.1093/molehr/gal036

Lee, K., Laviolette, S. R., & Hardy, D. B. (2021). Exposure to Δ9-tetrahydrocannabinol during rat pregnancy leads to impaired cardiac dysfunction in postnatal life. *Pediatric Research*, 1–8. https://doi.org/10.1038/s41390-021-01511-9

Lojpur, T., Easton, Z., Raez-Villanueva, S., Laviolette, S., Holloway, A. C., & Hardy, D. B. (2019). Δ9-Tetrahydrocannabinol leads to endoplasmic reticulum stress and mitochondrial dysfunction in human BeWo trophoblasts. *Reproductive Toxicology (Elmsford, N.Y.)*, *87*, 21–31. https://doi.org/10.1016/j.reprotox.2019.04.008

Maia, J., Midão, L., Cunha, S. C., Almada, M., Fonseca, B. M., Braga, J., Gonçalves, D., Teixeira, N., & Correia-da-Silva, G. (2019). Effects of cannabis tetrahydrocannabinol on endocannabinoid homeostasis in human placenta. *Archives of Toxicology*, *93*(3), 649–658. https://doi.org/10.1007/s00204-019-02389-7

Natale, B. V., Gustin, K. N., Lee, K., Holloway, A. C., Laviolette, S. R., Natale, D. R. C., & Hardy, D. B. (2020). Δ9-tetrahydrocannabinol exposure during rat pregnancy leads to symmetrical fetal growth restriction and labyrinth-specific vascular defects in the placenta. *Scientific Reports*, *10*(1), 544. https://doi.org/10.1038/s41598-019-57318-6

Neradugomma, N. K., Drafton, K., Mor, G. G., & Mao, Q. (2019). Marijuana-derived cannabinoids inhibit uterine endometrial stromal cell decidualization and compromise trophoblast-endometrium cross-talk. *Reproductive Toxicology*, *87*, 100–107. https://doi.org/10.1016/j.reprotox.2019.05.064

Oke, S. L., Lee, K., Papp, R., Laviolette, S. R., & Hardy, D. B. (2021). In Utero Exposure to Δ9-Tetrahydrocannabinol Leads to Postnatal Catch-Up Growth and Dysmetabolism in the Adult Rat Liver. *International Journal of Molecular Sciences*, *22*(14), 7502. https://doi.org/10.3390/ijms22147502

Singh, S., Filion, K. B., Abenhaim, H. A., & Eisenberg, M. J. (2020). Prevalence and outcomes of prenatal recreational cannabis use in high-income countries: A scoping review. *BJOG: An International Journal of Obstetrics and Gynaecology*, *127*(1), 8–16. https://doi.org/10.1111/1471-0528.15946

Walker, O. S., Ragos, R., Gurm, H., Lapierre, M., May, L. L., & Raha, S. (2020). Delta-9-tetrahydrocannabinol disrupts mitochondrial function and attenuates syncytialization in human placental BeWo cells. *Physiological Reports*, *8*(13). https://doi.org/10.14814/phy2.14476
